# Tissue and Serum Biomarkers in Degenerative Aortic Stenosis-Insights into Pathogenesis, Prevention and Therapy

**DOI:** 10.3390/biology12030347

**Published:** 2023-02-22

**Authors:** Alkistis Kapelouzou, Styliani Geronikolou, Irene Lidoriki, Christos Kontogiannis, Loukas Kaklamanis, Loukas Tsourelis, Dennis V. Cokkinos

**Affiliations:** 1 Center for Clinical, Experimental Surgery and Translational Research, Biomedical Research Foundation of the Academy of Athens, 11527 Athens, Greece; 2 1st Department of Surgery, School of Medicine, National and Kapodistrian University of Athens, Laiko Hospital, 11527 Athens, Greece; 3 Department of Clinical Therapeutics, National and Kapodistrian University of Athens Alexandra Hospital, 11527 Athens, Greece; 4 Department of Pathology, Onassis Cardiac Surgery Center, 17674 Athens, Greece

**Keywords:** valvular heart disease, degenerative aortic valve stenosis, biomarkers, irisin, periostin, osteoglycin, IL-18, HMGB-1, PCSK9

## Abstract

**Simple Summary:**

We found higher levels of six biomarkers significantly involved in cardiovascular pathology, i.e., irisin, periostin, osteoglycin, interleukin 18, high mobility group box 1 and proprotein convertase subtilisin/kexin type 9 in the serum at the protein level, and in the tissue at both the protein and mRNA levels of patients with AS (N = 60). Higher levels of all factors were found in DAS patients’ serum than in normal C (Ν = 22). All biomarkers were seen in the aortic valve cusps with DAS, but no trace of PCR mRNA was found in the five transplantation valves.

**Abstract:**

Background and Aim. Degenerative Aortic Stenosis (DAS) is a common disease that causes substantial morbidity and mortality worldwide, especially in the older population. Our aim was to further investigate novel serum and tissue biomarkers to elucidate biological processes involved in this entity. Material and Methods. We evaluated the expression of six biomarkers significantly involved in cardiovascular pathology, i.e., irisin, periostin, osteoglycin, interleukin 18, high mobility group box 1 and proprotein convertase subtilisin/kexin type 9 in the serum at the protein level, and in the tissue at both the protein and mRNA levels of patients with AS (N = 60). Five normal valves obtained after transplantation from hearts of patients with idiopathic dilated cardiomyopathy were also studied. Serum measurements were also performed in 22 individuals without valvular disease who served as controls (C). Results. Higher levels of all factors were found in DAS patients’ serum than in normal C. IHC and PCR mRNA tissue analysis showed the presence of all biomarkers in the aortic valve cusps with DAS, but no trace of PCR mRNA was found in the five transplantation valves. Moreover, periostin serum levels correlated significantly with IHC and mRNA tissue levels in AS patients. Conclusion. We showed that six widely prevalent biomarkers affecting the atherosclerotic process were also involved in DAS, suggesting a strong osteogenic and pro-inflammatory profile, indicating that aortic valve calcification is a multifactorial biological process.

## 1. Introduction

The interest in degenerative aortic stenosis (DAS) remains unabated, since it is an increasingly frequent cause of cardiac morbidity and mortality [1,2]. In a U.S. population study, its prevalence was 2.8% for peoples aged > 75 years [3]. It is correlated to atherosclerotic vascular disease, with many factors being prevalent in both conditions [1,2]. However, Ortlepp et al. [4] point out, that the predictive power of risk factors is not equal for the two entities. It is currently being accepted that DAS is not a passive process, but involves many mechanisms, such as lipoprotein profile derangement, oxidation, inflammation and valvular cell apoptosis [5,6,7]. All these are compounded by hemodynamic factors, since the initial valve sclerosis causes flow turbulence and nonlinear flow promoting further progress of the sclerosis/calcification process [1,6].

Livia Passos et al. [8] argue that cardiovascular calcification is an inflammatory disease, through crosstalk between innate and adaptive immune cell components.

A great number of biomarkers has been studied in DAS.

The purpose of our study is to identify additional serum and tissue biomarkers involved in patients with DAS, and also to investigate the correlation between their tissue and serum levels. Since 2015 we have been investigating tissue and serum biomarkers in this entity. We have shown that patients with DAS express more pro-inflammatory, calcification, fibrosis, proliferation and apoptosis biomarkers. We have also shown that Toll-like receptors and interleukin-37 are differentially expressed in aortic compared to mitral valves, indicating a higher pro-calcific and pro-inflammatory process in the aortic valve [7], in addition to the hemodynamic factors and the turbulent aortic flow.

Trying to obtain further insight in the calcification process we measured six biomarkers in the tissue of stenotic aortic valves excised at surgery for aortic valve replacement and compared them to normal aortic valves obtained at cardiac transplantation.

These six biomarkers were also measured in the serum of patients and controls without any cardiovascular disease; the reasons for their inclusion are explained below.

### 1.1. Irisin Levels

The myokine irisin, which is cleaved from the plasma membrane FNDC5, is more highly expressed in cardiac muscle than in skeletal [9]. It is also highly elevated in patients with heart failure and preserved ejection fraction (EF) more than in those with reduced EF; in the former, it correlated with total antioxidant capacity [10]. Irisin is increased in acute heart failure and is an independent factor for 1 year all-cause mortality [11]. However, it is decreased in myocardial infarction, both clinical and experimental [12,13]. It is correlated negatively to coronary artery disease severity [14]. Patients in the higher SYNTAX score levels were older and had lower irisin levels than younger ones. However, it has also been reported to be cardioprotective [15].

Irisin has been found to promote osteoblast proliferation and differentiation via the MAP kinase pathways by Qiao et al. [16] who postulated its possible use in osteopenia. It increases sclerostin expression in osteocytes to induce bone resorption. It mediates this effect via αV integrin receptors [17]. In our previous study we have found sclerostin to be increased in DAS [5]. Irisin administration has been proposed for treatment of osteoporosis by Colaioanni et al. [18].

### 1.2. Periostin

Hakuno et al. [19] found PN to be increased in degenerative or rheumatically affected heart valves. The same authors also found that in wild type mice a high fat diet markedly increased its expression in both AV and MV together with the fibrotic markers MMP2 and MMP13. It is associated with myocardial fibrosis in human heart failure [20]. It was increased together with MMP2 activity. It is increased in hypertrophic mice hearts together with interstitial fibrosis [21]. It decreases together with a reduction in myocardial fibrosis in hearts unloaded both clinically (LVAD) and experimentally in mice (aortic arch de-banding) [22]. It is also a potential marker biomarker for coronary artery disease with acute heart failure [23]. Furthermore patients with CAD had higher levels than controls at similar ages (around 63 y).

### 1.3. Osteoglycin

The same pattern for PN was found for OGN, which is implicated in matrix homeostasis. It modulates fibrosis and inflammation. Deckx et al. [24] found that its levels were higher in patients with AS with less severe myocardial fibrosis, in whom it was negatively correlated with collagen content in the myocardium, but they did not measure it in the valves. Van Aelst et al. [25] found that it prevents cardiac dilation and dysfunction after myocardial infarction through infarct collagen strengthening. Zuo et al. [26] have found that osteoglycin attenuates cardiac fibrosis; it could be an antifibrotic, but is also pro-calcific by suppressing myofibroblast proliferation. Circulating osteoglycin and NGAL/MMP9 complex concentrations predict 1 y MACE after coronary arteriography [27]. It was statistically slightly higher in CAD patients aged 70 vs. 65 y [28] Tanaka et al. have stated that it is a humoral bone anabolic factor [28]

### 1.4. Interleukin 18

IL-18 is a dominant pro-inflammatory cytokine. In the heart, it is produced by infiltrating neutrophils, resident macrophages, endothelial cells, smooth muscle cells and cardiomyocytes [29]. In the non-rheumatic aortic valve, increased tissue levels have been found as compared to controls [30], and are correlated to advanced clinical severity. It promotes myofibroblast activation of porcine valvular interstitial cells [31]. Interestingly, the administration of increased doses of IL-18 upregulated the expression of osteopontin [32] which we have found to be pro-osteogenic [5]. It is prospectively and independently associated with CVD risk [33] in patients of similar ages (52.5 y).

### 1.5. HMGB1

The high mobility group box 1 (HMGB1) which is also a pro-inflammatory factor has been implicated in the pathogenesis of DAS. It has been found to be increased in the calcific regions; the same was found in regard to TLR by Shen et al. [34], who postulate that TLR4 may function as an essential mediator of HMGB1-induced calcification and in the activation of p38 and NFkB. Wang et al. [35] found that HMGB1 protein and TLR4 are upregulated in vitro by HMGB1 in aortic valvular interstitial cells. We have described in detail the role of TLRs in aortic and mitral valve stenosis [7]. Its increase in the serum is related to severity of coronary artery stenosis [36].

### 1.6. PCSK9

Recent experimental studies have demonstrated that PCSK9 might directly promote inflammation, apoptotic cell death and endothelial dysfunction in the atherosclerotic process and plaque formation [37] and is associated with the severity of CAD [38] in hypercholesterolemia, cardiovascular inflammation and diabetes [39,40,41].

Wang et al. [42] and Poggio et al. [43] found lower calcium content in aortic valves of PCSK9^−^/^−^ mice than wild type animals. The former were resistant to production of aortic calcification with two pro-calcification diets (β-glycero–phosphate and ascorbic acid). The same authors found that human calcified aortic valves expressed higher PCSK9 than non-calcified ones. Interestingly, Salaun et al. [44] found that elevated plasma levels of PCSK9 were a risk factor for hemodynamic deterioration of surgically implanted bioprosthetic aortic valves. The same held true for LP-PLA2 and HoMA. PCSK9, apart from its action on LDL receptors, can promote apoptotic cell death of neurons [45].

## 2. Material and Methods

### 2.1. Ethics Statement

This study was approved by the Ethics Committees of the Onassis Cardiac Surgery Center (OCSC) and conformed to the principles outlined in the Declaration of Helsinki. Written informed consent was obtained from all patients.

### 2.2. Study Population

The present, prospective, open-label study, extends the results of our previously published studies [5,7]. The age of the 60 DAS patients was 66.1 ± 12.5 years, 50% were women. Echocardiography was obtained in all patients. Aortic valve area was 0.9 to 0.5 cm [2]. Patients were taking antihypertensive (75%) and antilipidemic (80%) drugs during the last two years. However, none had significant coronary artery disease necessitating concomitant aortocoronary bypass surgery. Patients with rheumatic cardiac disease, bicuspid aortic valve or connective tissue disorders were excluded. The excised valve cusps were harvested during surgery. Blood was collected from AS patients before valve surgery and from twenty-two (22) healthy subjects without any chronic cardiovascular or metabolic disease, and not receiving any long-term medication, who served as healthy control group (C), for comparison of serum biomarkers; mean age was 34.4 ± 7.5 years, 50% were women. None were steadily employing radioactive drugs.

We also obtained 5 aortic valves from patients undergoing cardiac transplantation, 3 men and 2 women (min age 48.4), because of terminal heart failure due to idiopathic dilated cardiomyopathy, who did not show any sclerotic or calcific aortic valve changes.

### 2.3. Blood Analysis

Blood samples were collected by venipuncture after subjects were fasted overnight. Serum was collected and stored at −80 °C, analysis was performed in duplicate; dilution was assessed as per protocol. We used commercially available enzyme immunoassay kits for irisin (FNDC5) (EK-067-29, Phoenix Pharmaceuticals, California, CA, USA); periostin (PN) (EHPOSTN, Thermoscientific, Germany); osteoglycin (OGN) (CSB EL016314HU, Cusabio, Houston, TX, USA); IL-18 (DY318-05, R&D, Minneapolis, MN, USA); high-mobility group box 1, HMGB1 (LS-F11641, Lifespan Ltd., Seattle, DC, USA); PCSK9 (DPC900, R&D, Minneapolis, MN, USA) and quantified each protein according to the protocol of the manufacturer with an ELISA reader system (Spectramax 190; Molecular Devices, Sunnyvale, Calif, CA, USA).

### 2.4. Valve Cusp Immunohistochemistry and Quantitative Morphometrical Analysis

Aortic valve cusps were excised and one part of each valve tissue was placed in a container for immunohistochemistry (IHC) analysis at the pathology department of the OCSC and the Biomedical Research Foundation of Academy of Athens according to our previous protocol. The protocol of IHC has been described and validated in our lab [46]; FNDC-5 (PA5-62368, 5 μg/mL, Invitrogen, CA, USA); PN (PA5-82458, 5 μg/mL, Invitrogen, CA, USA); OGN (PA5-48255, 5 μg/mL, Invitrogen, CA, USA); IL-18 (PA5-79479, Invitrogen, CA, USA) HMGB1 (PA5-80691, 5 μg/mL, Invitrogen, CA, USA); PCSK9 (PA5-79789, 5 μg/mL, Invitrogen, CA, USA) were used for IHC. IHC was performed according to the manufacturer’s protocol by using the development kit (Zytochem Plus; Zytomed system, Germany). Appropriate isotype negative controls were performed at the same concentrations as the primary antibodies. Microscopic investigation of the IHC sections was performed with stereology upright Leica DMRA2 camera, and were analyzed by stereo-investigator 10 program (version 10.1, MBF Bioscience, Microbrightfield. Inc., Willinston, VT, USA) in order to quantify the extent of the tissue covered by each antibody.

### 2.5. RNA Isolation and qRT PCR Analysis

Total RNA was extracted using Trizol reagent (Sigma, Saint Louis, MO, USA) according to the manufacturer’s instructions [46]. The RNA quality was assessed with agarose gel electrophoresis and quantitated spectrophotometrically. cDNA was synthesized by RT (MMLV, reverse transcriptase; Sigma), and real-time quantitative polymerase chain reaction was performed by using SYBR Green (Invitrogen, Life Technologies, New York, NY, USA). The primers synthesized by Origine (Herford, Germany) were used as documented in Table 1. The thermal cycling protocol was performed according to our lab protocol [5,7].

We also measured the 6 biomarkers already discussed in the 5 aortic valves explanted from patients who had undergone cardiac transplantation by RNA isolation and qRT analysis.

All had idiopathic dilated cardiomyopathy without coronary artery disease at arteriography: 2 were women aged 28 and 56 years, and were 3 men aged 48, 54 and 58 years.

### 2.6. Statistical Analysis

Shapiro–Wilks test for normality showed that none of the variables had normal distribution. Thus, Univariate and Multivariate analysis, one-way ANOVA or *t*-test were inappropriate tests for the analysis. We performed non-parametric tests instead. The Mann–Whitney test for evaluating the patients versus control serum biomarkers showed significant differences between all serum markers. All correlations were performed with non-parametric Spearman’s rho. Alpha was set at 0.05. Statistics were performed with SPSS28.

## 3. Results

### 3.1. Serum Findings

**Figure 1:**
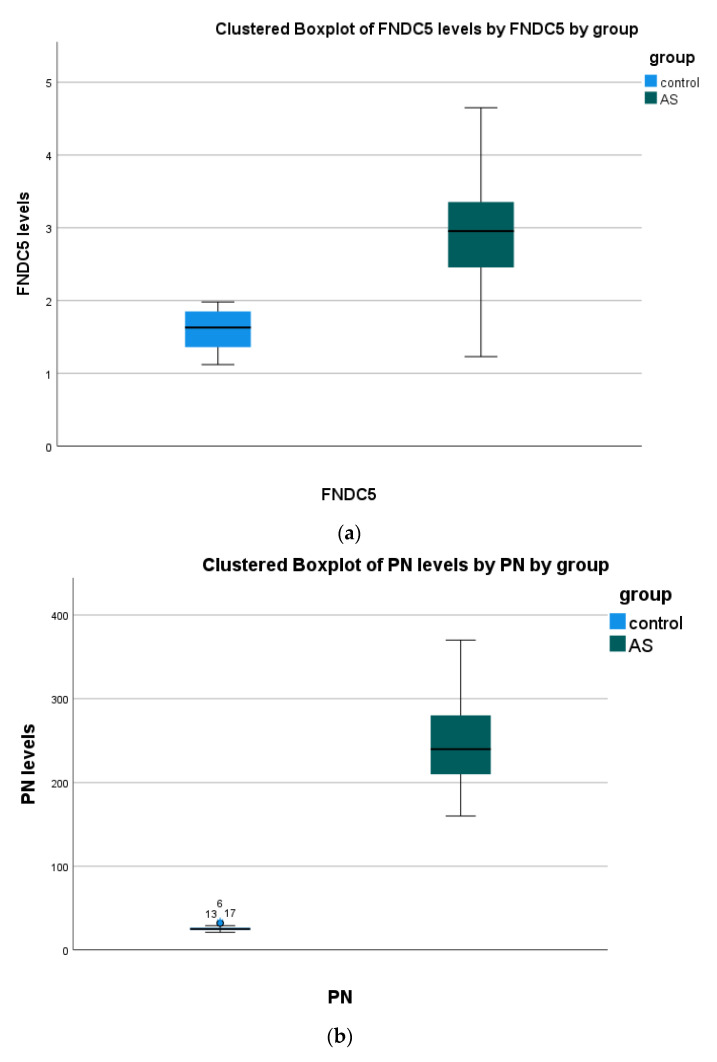

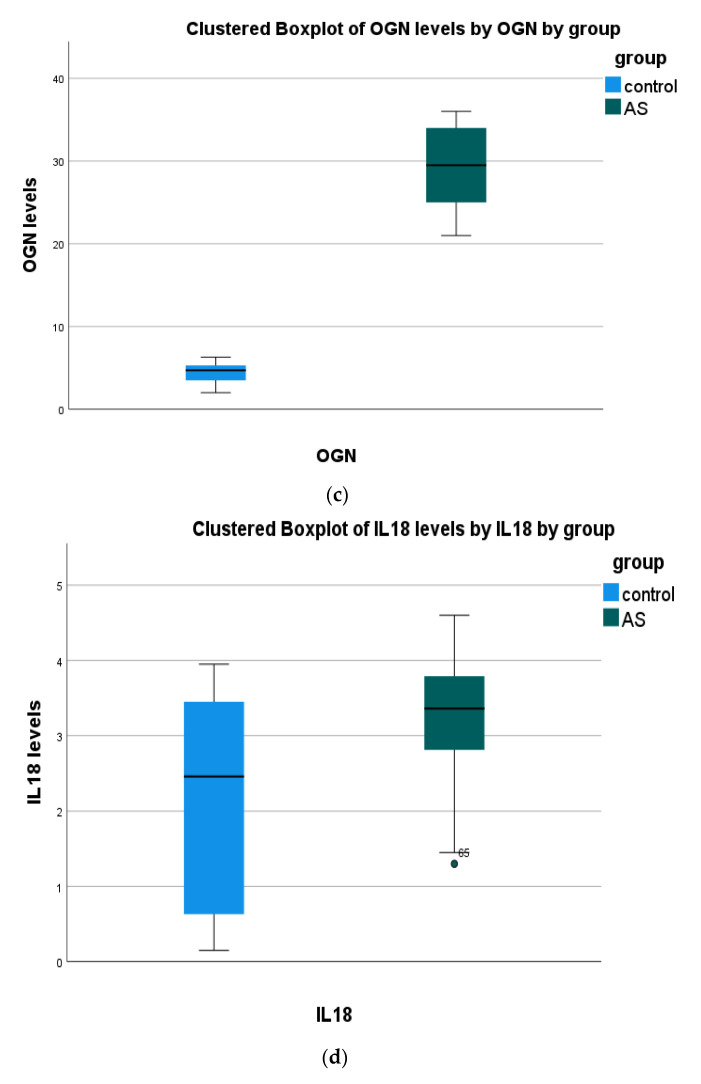

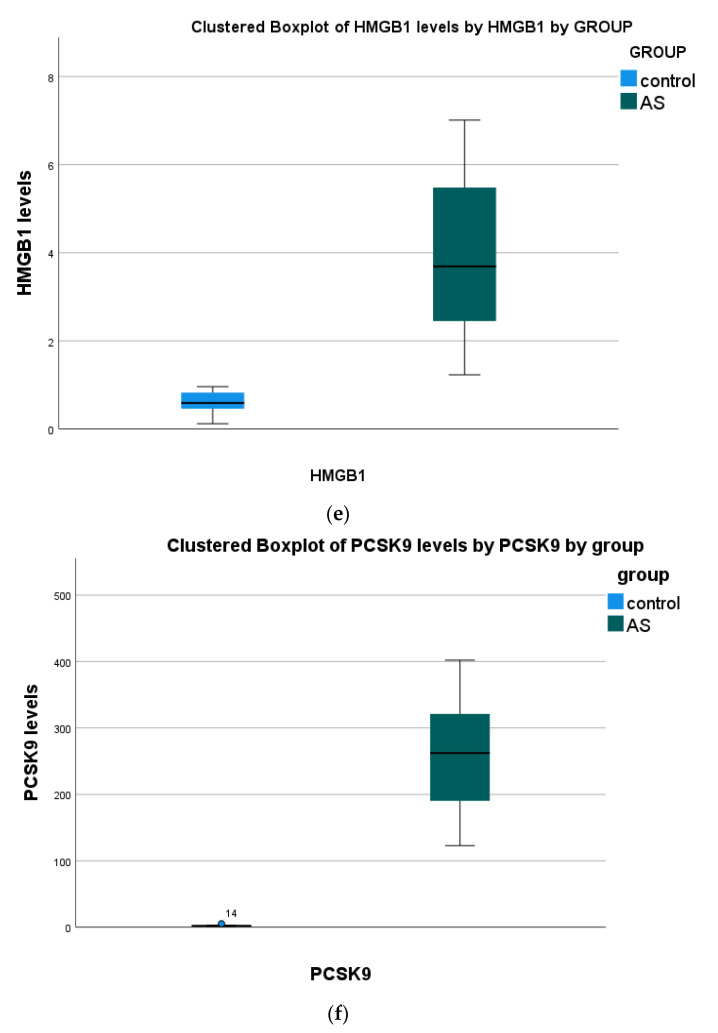
Serum biomarkers’ concentration in all study groups. Data presented as boxplots: Blue color, Control; Green color, (AS) aortic stenosis patients. Abbreviations: (**a**) irisin (FNDC5) (**b**) periostin (PN); (**c**) osteoglycin (OGN); (**d**) interleukin 18 (IL-18); (**e**) high-mobility group box 1 (HMGB1); (**f**) proprotein convertase subtilisin kexin 9 (PCSK9).

(a) The non-parametric Mann–Whitney test showed significant differences in all serum biomarkers between patients and the control group. (Figure 1, Table 2).

(b) Positive correlations were found in HMGB1 with PCSK9 and PN vs. PSCK9. Negative correlations were observed in OGN with PN and IL-18.

Any other correlation was found insignificant (*p* > 0.05).

(c) Positive correlations were found in HMGB1 with PCSK9 and PN with PSCK9. Negative correlations were observed in OGN with PN and IL-18.

(d) Any other correlation was found insignificant (*p* > 0.05).

### 3.2. Tissue vs. Serum

(a) Of all biomarkers found significantly correlated only PN tissue levels correlate with the same marker’s levels detected in serum.

(b) For all other tissue markers described in Table 3, the correlations were found in non-identical markers: (positive) tissue HMGB1 with serum OGN, tissue PCSK9 with serum OGN, (negative) tissue IL-18 with serum PCSK9 and OGN, tissue HMGB1 with serum IL-18, tissue PCSK9 with serum HMGB1 and IL-18. (Table 3)

(c) The insignificant correlations were not included in Table 3.

### 3.3. Immunohistochemistry Biomarkers in Aortic Valve Cusps

**Figure 2:**
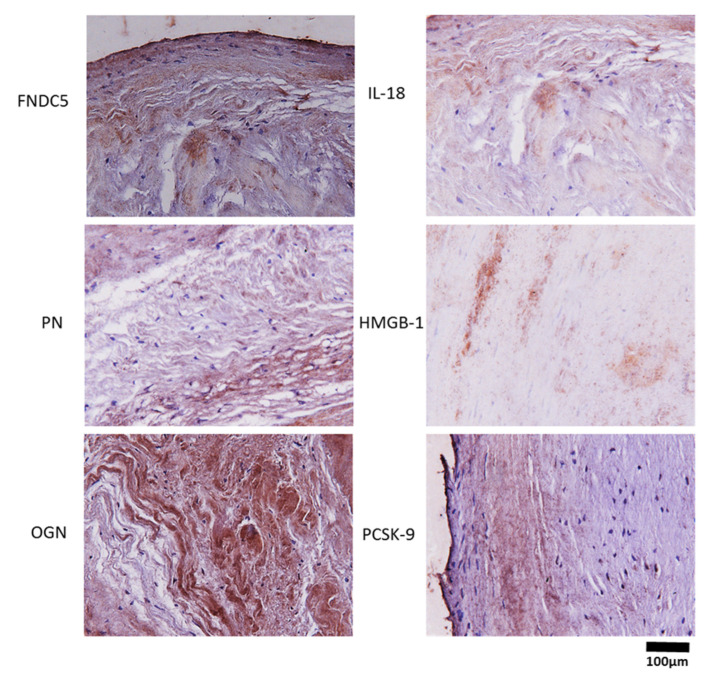
Immunohistochemistry staining in the AS group. Representative photos of tissue biomarkers from aortic valve cusps. Nuclei stained with celestine blue are shown in blue, expression of biomarkers shown in brown. 40× magnification. Abbreviations: irisin (FNDC5); periostin (PN); osteoglycin (OGN); interleukin 18 (IL-18); high-mobility group box 1 (HMGB1); proprotein convertase subtilisin kexin 9 (PCSK9).

Immunohistochemistry staining was performed (Figure 2). Biomarker tissue presence was confirmed in all AVC samples.

### 3.4. mRNA Expression of Inflammation and Calcification Biomarkers in AS Patients

**Figure 3:**
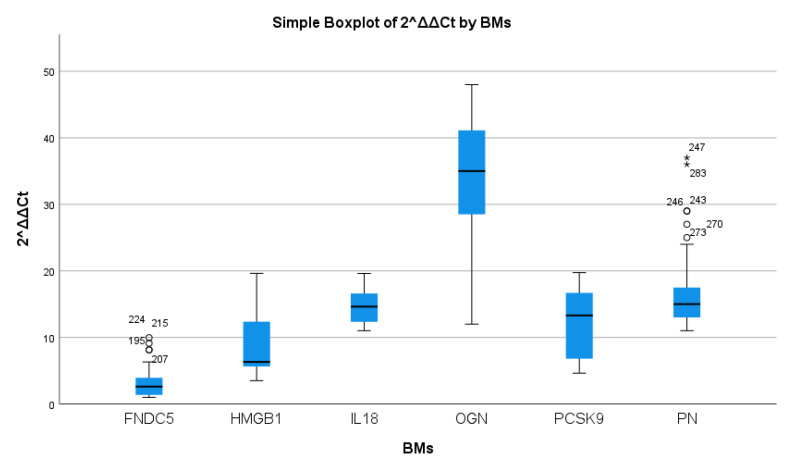
mRNA expression of tissue biomarker concentrations in AS patients. Data presented as boxplots; Abbreviations: irisin (FNDC5); periostin (PN); osteoglycin (OGN); interleukin 18 (IL-18); high-mobility group box 1 (HMGB1); proprotein convertase subtilisin kexin 9 (PCSK9). Non-normality confirmed by outliers imposed non-parametric evaluation opted herein.

Tissue mRNA levels of all biomarkers was present in AVC (Figure 3).

The highest expression was found for osteoglycin, while FNDC5 was only mildly elevated

None of the valves from patients with cardiac transplantation had any expression of mRNA of the above biomarkers.

The five valves from the cardiac transplantation hearts showed no expression at all.

### 3.5. Tissue Biomarkers Correlations

We found significant correlations between most tissue biomarkers.

## 4. Discussion

In this study we continue the investigation of the calcification process through the novel body of biomarkers examined in DAS [5,7]. We investigated six factors, both in serum and valve tissue, of which some have been very scantily studied. All of them have also been involved in CAD. Thus, we did not consider it practical to add a comparison Group with CAD only and without DAS.

We obtained valves from five patients undergoing cardiac transplantation, which did not show any expression of RNA of these biomarkers. With regard to the serum biomarkers, it must be realized that the control individuals were younger. However, this difference reflects a real-life situation, that individuals develop DAS later in life. It must be realized that DAS and CAD co-exist in around 50% of patients, as presented by Ortlep et al. [4]. However, none of our patients needed any concomitant surgery for CAD, thus excluding significant disease.

Our study may offer another biomarker, periostin, which may in the future provide prognostic information. Up to now, NT-proBNP [47] and BNP [48] have provided data with regard to intervention time because they reflect myocardial stress which would prompt information. A valve-specific marker such as periostin may provide means of following the course of sclerosis to stress.

Again, we must stress that we did not perform a population study so as to assess the importance of these biomarkers for predicting the presence of DAS.

Νew technologies to measure early calcification and inflammation are available. Dweck et al. have provided data from position emission tomography in vivo [49] in patents with DAS.

It must be noted that we did not find any microscopic evidence of changes in the normal aortic valves obtained at cardiac transplantation, nor by mRNA PCR, which is more reliable than immunohistochemistry [50].

Our findings confirm the association of PCSK9 with valve tissue calcification, and supports the postulations that PCSK9 inhibitors or drugs preventing its production by the liver could be a consideration for prevention of the course towards AS once aortic valve sclerosis has been diagnosed. In fact, in the FOURIER study, PCSK9 inhibition was associated with a lower hazard of new or worsening stenosis AS [51]. This is especially relevant since initial trials with statins have not been successful in preventing AS [52,53]. While there was some promise in a rosuvastatin trial with echo measurements [54], this was not validated in the ASTRONOMER TRIAL [55]. A reason might be that statins do lower cholesterol but increase PCSK9 levels [56]. Thus, well-controlled therapeutic trials with PCSK9 inhibitors are warranted.

In the coronary arteries statins actually promote calcification, while decreasing atherosclerotic plaque burden and fibrosis [57]. This action may explain their lack of influence on the progression of aortic calcification. We found that the PCSK9 receptor is strongly expressed in the aortic valve both by IHC and mRNA analysis and increased in the serum as compared to controls. This supports the population study of Perrot et al. [58] who found that AS was less frequent in carriers of the PCSK9 R461 loss-of-function variants. They also found increased expression of PCSK9 in calcified human valves.

The high interrelation between PCSK9 and aortic valve calcification has already been stressed [59]. Additionally, this family of drugs antagonizes apoptosis; [45,56] endothelial apoptosis [60] is found in endothelial cells in DAS.

PCSK9 inhibitors alleviate oxidation and inflammation [61].

Another candidate drug family would be SGLT2 inhibitors. Interestingly, they have been found to attenuate the secretion of IL1β and IL-18 [59] by repressing the HMGB1-TLR4 receptor axis [62]. They also antagonize many inflammatory interleukins [63,64,65]. They are also considered powerful antioxidants [66]. Possibly, if these families of drugs for PCSK9 and SGLT2 are given together they could exert a synergistic effect.

Another practical aspect of our finding is if serum levels could predict or follow the course of aortic valve sclerosis towards actual stenosis, by a set of easily measured biomarkers. This held true for periostin in our patients.

We tried to address the mechanisms of DAS. The fact that none of these biomarkers are found in the tissue of normal aortic valves in control patients undergoing cardiac transplantation of a similar age to those with AVR suggests that this is not a problem regulated only by age. As it regards serum levels, all our control patients had much lower levels than those with DAS, which is not surprising. Furthermore, biomarkers in DAS are legion and concern all aspects of inflammation, oxidative stress, pro-calcification effects and lipid metabolism.

It should also be stressed that they all have been associated with CAD as well, since approximately half of the patients with DAS have CVD as well. As already stated, coronary artery disease is strongly associated with DAS in many studies considering the age of our patients; its prevalence could be estimated between 41% and 51% [4,67].

Indeed, non-obstructive aortic valve calcification has become a window into significant coronary artery disease [68].

However, since as already stated, many biomarkers are being studied, we did not address any other correlations in the tissue which would create confusion, since it would be difficult to find practical explanations and promote far-fetched postulation.

We believe that our findings add impetus to the efforts towards preventing the progression of aortic sclerosis to frank DAS by drugs affecting causative factors, such as hyperlipidemia, inflammation, oxidation and endothelial apoptosis. This is a realistic goal in parallel to CAD, where, although interventional and surgical therapies have attained excellent results, efforts at prevention continue unabated.

## 5. Study Limitations

The lower serum levels of most markers in our controls may be because they are of younger age and there is an absence of comorbidities. However, we do not consider that we have a new diagnostic and pragmatic biomarker, but we have investigated the pathobiology of this entity.

## 6. Conclusions

Patients with stenotic aortic valves express higher pro-inflammatory, calcification, fibrosis, proliferation and apoptosis-expressing markers in their serum than normal controls. They are all strongly expressed in calcified, but not normal, valves. We found that PN concentration is an important finding that can lead us to a consideration of the prognostic role of serum biomarkers in the course of this pathological process. Our findings point toward a higher pro-calcification and pro-inflammatory profile in DAS patients. We believe that our findings provide interesting data for the diagnosis and prevention of aortic sclerosis, and possibly treatment of mild AS.

## Figures and Tables

**Table 1:** Primers sequences. Abbreviations: Irisin (FNDC5); periostin (PN); osteoglycin (OGN); interleukin 18 (IL-18); high-mobility group box 1 (HMGB1); proprotein convertase subtilisin kexin 9 (PCSK9).

Primers	Forward	Reverse	bp	NM
b actin	CACCATTGGCAATGAGCGGTTC	AGGTCTTTGCGGATGTCCACGT	101	1101
FNDC5	AGCGAGCCTGTGCTCTTCAAGA	GAACAGGACCACGACGATGATC	120	1171940
PN	CAGCAAACCACCTTCACGGATC	TTAAGGAGGCGCTGAACCATGC	118	1135934
OGN	CCATAATGCCCTGGAATCCGTG	CAGGCGTATCTCTTCAATGCGG	125	14057
IL-18	GATAGCCAGCCTAGAGGTATGG	CCTTGATGTTATCAGGAGGATTCA	120	1243211
HMGB-1	GCGAAGAAACTGGGAGAGATGTG	GCATCAGGCTTTCCTTTAGCTCG	114	1313892
PCKS-9	GACACCAGCATACAGAGTGACC	GTGCCATGACTGTCACACTTGC	118	174936

**Table 2:** Statistical analysis of serum biomarkers in all study groups. Mann–Whitney test for all markers measured in the serum of both groups (control and AS); alpha was set at 0.05 C and AS. Abbreviations: C (control); AS (aortic stenosis patients); Irisin (FNDC5); periostin (PN); osteoglycin (OGN); interleukin 18 (IL-18); high-mobility group box 1 (HMGB1); proprotein convertase subtilisin kexin 9 (PCSK9).

Serum Markers	Median Control [95% CI]	Median AS [95% CI]	Mann–Whitney U	*p*-Value
IL-18	2.46 [1.26, 3.02]	3.36 [2.95, 3.56]	259.5	0.004
HMGB1	0.59 [0.44, 0.76]	2.98 [2.4, 13.86]	0.00	<0.001
PCSK9	2.05 [1.2, 82.86]	262.00 [1.57, 224.286]	0.00	<0.001
OGN	4.7 [3.5, 29.78]	29.5 [27.64, 30.9]	0.00	<0.001
PN	25.00 [23.28, 45.75]	240.00 [227.54, 265.36]	0.00	<0.001
FNDC5	1.63 [1.1, 43.74]	2.955 [2.2, 3.73]	31.00	<0.001

**Table 3:** The Spearman’s rho coefficients, *p*-value and 95% Confidence Intervals of every significant correlation between and within the tissues under investigation.

	Biomarkers Compared	Spearman Rho	*p*-Value	95% Confidence Intervals
Serum vs. serum	OGN with PN	−0.359	0.005	[−0.567, 0.108]
	IL-18 with OGN	−0.441	<0.001	[−0.630, 0.203]
	HMGB1 with PCSK9	0.445	0.001	[0.173, 0.610]
	PN vs PSCK9	0.286	0.027	[ 0.027, 0.509]
Tissue vs. tissue	IL-18 with HMGB1	−0.425	<0.001	[−0.618, −0.185]
	IL-18 with PCSK9	−0.528	<0.001	[−0.694, −0.310]
	HMGB1 with PCSK9	0.420	0.001	[0.178, 0.613]
Tissue vs. serum	IL-18 with FNDC5	−0.291	0.024	[−0.513, −0.033]
	IL-18 with OGN	−0.419	0.001	[−0.613, −0.177]
	HMGB1 with IL-18	−0.326	0.011	[−0.541, −0.071]
	HMGB1 with OGN	0.282	0.029	[0.022, 0.506]
	PCSK9 with IL-18	−0.405	0.001	[−0.602, −0.161]
	PCSK9 with HMGB1	−0.326	0.011	[−0.541, −0.070]
	PCSK9 with OGN	0.604	<0.001	[0.407, 0.747]
	PN with PN	0.289	0.025	[0.030, 0.511]

## Data Availability

The data is not available due to privacy or ethical restrictions.

## Abbreviations

AS, aortic stenosis; AVC, aortic valve cusp; CAD, coronary artery disease; CVD, cardiovascular disease; FNDC5, irisin; PN, periostin; OGN, osteoglycin; IL-18, interleukin 18; HMGB-1, high mobility group box 1; IHC, immunochemistry; PCSK9, proprotein convertase subtilisin/kexin type 9; qRT-PCR, quantitative reverse transcription polymerase chain reaction.
